# DDX59 promotes DNA replication in lung adenocarcinoma

**DOI:** 10.1038/cddiscovery.2016.95

**Published:** 2017-01-09

**Authors:** Jin You, Xingshun Wang, Jiuling Wang, Baolei Yuan, Yandong Zhang

**Affiliations:** 1 Department of Biology, Southern University of Science and Technology, Shenzhen, Guangdong, China

## Abstract

DEAD box proteins are multifunctional proteins involved in every aspect in RNA metabolism and have essential roles in many cellular activities. Despite their importance, many DEAD box proteins remain uncharacterized. In this report, we found DDX59 overexpressed in lung adenocarcinoma. DDX59 knockdown reduced cell proliferation, anchorage-independent cell growth, and caused reduction of tumor formation in immunocompromised mice. In multiple lung cancer cells, we found that DDX59 knockdown inhibits DNA synthesis; wild-type DDX59 but not helicase-defective mutant of DDX59 enhances DNA synthesis. DDX59 knockdown caused reduction of MCM protein levels, decreased the loading of MCM ring protein onto chromatin, and therefore inhibited DNA replication. Our study reveals for the first time that DDX59 has an important role in lung cancer development through promoting DNA replication.

## Introduction

DEAD/DEAH box RNA helicases are highly conserved from lower organisms to higher organisms.^1,2^ They are characterized by a DEAD/DEAH box and seven other consensus sequences in their primary amino-acid sequences.^3^ They have diverse and important roles in almost every aspect of RNA metabolism, such as ribosome biogenesis, miRNA biogenesis, transcription, splicing, translation, and mRNA decay.^4–6^ Several recent studies show that RNA helicases control a few important signaling pathways including Wnt, Notch, and Estrogen Receptor signaling.^7–9^ DEAD box proteins have also been found to participate in DNA replication and genomic stability.^10–12^ A recent whole-genome screening through CRISPR gene editing found many DEAD/DEAH box proteins to be essential in promoting cancer cell proliferation and survival.^13,14^ More and more publications reveal the deregulation of these proteins in various human cancers.^9,15–18^

Lung cancer causes the most cancer-related death worldwide;^19^ it can be divided into small cell lung cancer and non-small cell lung cancer (NSCLC), which takes 85% of all cases.^20^ Comprehensive cancer genome sequencing found both genetic and epigenetic alterations in NSCLCs.^21–23^ Alterations in key genes, such as *Ras*, *ALK* and *EGFR*, have previously been shown to drive lung tumorigenesis.^24–27^ Although targeted therapy has been developed and utilized widely to treat EGRF and ALK mutant lung cancer, many other lung cancers remain poorly characterized.

Through a data portal platform for TCGA database (www.cbioportal.org), we evaluate a DEAD box protein, DDX59, whose function has not been characterized before.^28^ We found that *DDX59* gene is frequently amplified in many human cancers. For instance, in human liver cancer, it is amplified at a percentage of 14%; in breast cancer, it is amplified in 12% of all cases; in lung adenocarcinoma, it is amplified at a percentage of 8%.

We then analyzed the DDX59 protein levels in malignant tissues and normal human lung tissues. We found that DDX59 is highly expressed in lung adenocarcinoma tissues. Acute depletion of DDX59 protein caused cell cycle arrest. Cancer cells with DDX59 knockdown could not form tumor in xenograft model. Finally, we provide original evidence for the function of DDX59 in DNA replication. We found that DDX59 regulates MCM protein levels, and therefore promotes DNA synthesis.

## Results

### DDX59 is highly expressed in lung adenocarcinoma

**Figure 1:**
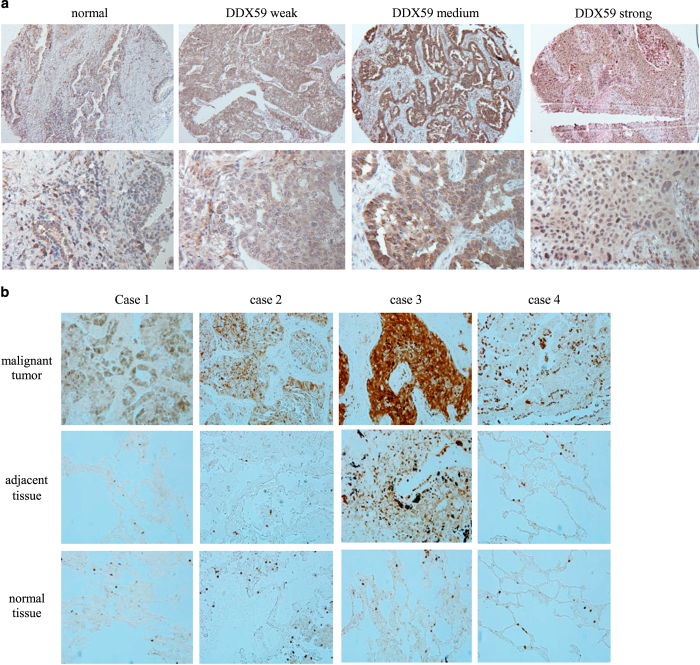
DDX59 is highly expressed in non-small cell lung cancers. (**a**) A non-small cell lung cancer tissue array was used for immunohistochemistry staining by anti-DDX59 antibody. Typical images are shown for normal and cancer tissues. DDX59 staining signals were categorized into weak, medium, or strong based on signal strength. Representative images are shown. (**b**) Totally, 33 pairs of lung adenocarcinoma, tumor adjacent normal tissues, and normal lung tissues were compared for DDX59 protein expression by IHC staining. Representative images are shown.

The DEAD box proteins have diverse roles in cellular activities; however, little is known about their roles and functions in cancers. We searched the TCGA database and found that DDX59 is amplified in ~9% of human lung adenocarcinoma cancers. *DDX59* gene is also amplified in several other cancer types including breast cancer, liver cancer, and melanoma. To investigate whether DDX59 protein is highly expressed in lung cancers, we first performed an immunohistochemistry (IHC) staining to detect DDX59 in a human NSCLC tissue array containing 95 cases of NSCLCs. As shown in Figure 1a, we found that approximately half of these tumor tissues showed positive staining for DDX59, whereas in three normal lung tissues, DDX59 was weakly expressed. Among all the subtypes of NSCLCs, positive staining for DDX59 was observed in most lung adenocarcinoma (Supplementary Table 1). To check whether DDX59 protein is indeed elevated in lung adenocarcinoma, we further performed IHC for 33 additional cases of human lung adenocarcinoma tissues with paired tumor adjacent normal and normal tissues. We found that DDX59 positively expressed in 56% of all cases in the tumor tissues, whereas most paired tumor adjacent and normal tissues show negative staining for DDX59 (Figure 1b; Supplementary Table 2). In lung adenocarcinoma, DDX59 protein expression does correlate with tumor stages (Supplementary Table 3). From these IHC results, it is clear that DDX59 localized mostly in the nucleus rather than in the cytosol of lung cancer tissues. Many tissues have primarily nuclear DDX59, whereas a few other tissues have both cytosolic and nuclear DDX59.

### DDX59 expression in lung cancer cell lines

**Figure 2:**
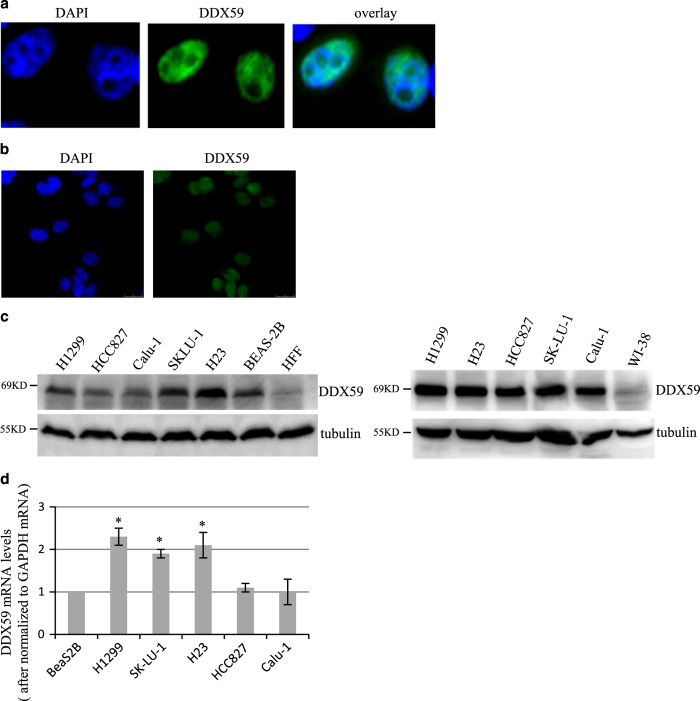
DDX59 expression in non-small cell lung cancer cell lines. (**a**) FLAG-DDX59 was transiently transfected into H1299 cells; cells were then fixed and stained with anti-FLAG (green). DAPI demarcated nuclei. (**b**) H1299 cells were fixed and stained with DDX59 antibody (red). DAPI was used to stain nuclei (blue). (**c**) A panel of non-small cell lung cancer cell lines and normal lung cell lines was analyzed by western blots for DDX59 protein levels, with tubulin as an internal control. (**d**) The cells were then analyzed for total levels of DDX59 mRNA. Error bars are calculated from three independent analyses. *P<0.05, *n*=3.

To characterize this novel protein in cells, we first analyzed the cellular localization of DDX59. We cloned a full length of DDX59 consisting of 619 amino acids according to the sequence provided by Ensembl (www.ensembl.org). We then expressed this protein in lung cancer cells and found DDX59 predominantly expressed in the nucleus (Figure 2a). We further performed immunofluorescence for endogenous DDX59 and found it to localize in the nucleus, too (Figure 2b). To check DDX59 protein levels in cancer cell lines, we analyzed several different lung cancer cell lines and the immortalized human lung epithelial cell line, BeaS2B, as well as normal lung cell line, WI-38, primary human fibroblast, HFF. As shown in Figure 2c, we found DDX59 highly expressed in many lung cancer cell lines as compared with normal lung cells. We further analyzed the mRNA levels of DDX59 in these cell lines. Three lung cancer cell lines, including H1299, H23, and SK-LU-1, contain higher levels of DDX59 mRNA as compared with normal lung cell lines by up to twofold (Figure 2d).

### DDX59 is important to maintain cancer cell phenotype

**Figure 3:**
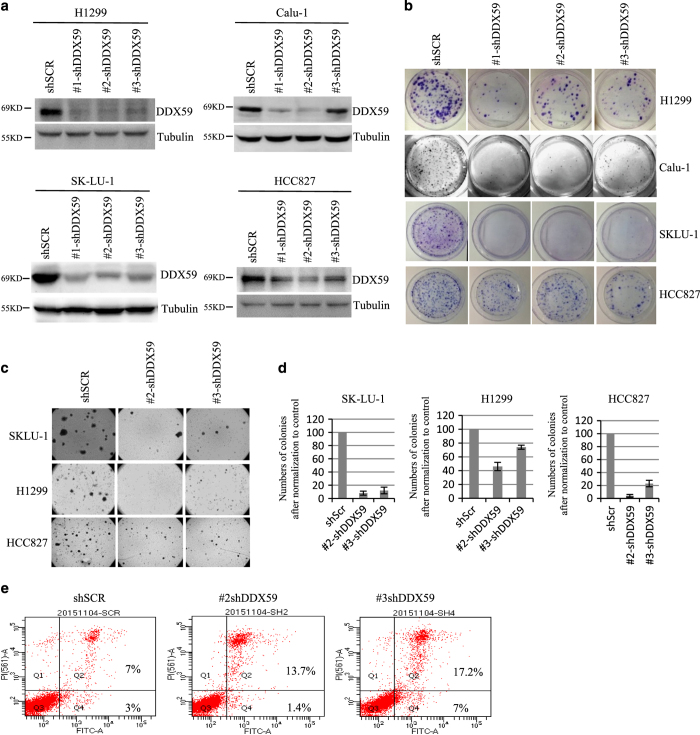
DDX59 is important to maintain cancer cell phenotype. (**a**) Calu-1, SK-LU-1, HCC827, and H1299 lung cancer cells were either infected with shSCR or shRNA-DDX59 lentivirus. Four days post lentiviral infection, cells were collected. Whole-cell lysates were subjected to western blot analysis with anti-DDX59 and anti-tubulin antibodies. (**b**) Three days post infection, cells were split and ~5000 cells per condition were plated on 100-mm dish in triplicates. Cells were further cultured for 2 weeks until being subjected to Foci analysis. Typical images for the culture of the indicated transduced cells are shown. (**c**) SK-LU-1, HCC827, and H1299 cells were transduced with either shSCR or shRNA-DDX59 lentivirus. Cells were subjected to soft-agar analysis after plating 1×10^4^ cells into each 60-mm dish. Typical image is shown after culturing for 2 weeks. (**d**) Quantitation of colony numbers in the soft-agar assay is graphed; error bars were taken from triplicates. *P<0.05, *n*=3. (**e**) Above-mentioned cells were subjected to flow cytometry analysis after staining with Annexin V and PI using the Vybrant apoptosis kit. Percentage of apoptotic cells were labeled in the quadrants.

To study the function of DDX59 in cells, we designed several different shRNAs to knockdown DDX59. As shown in Figure 3a, all of these shRNAs could knockdown DDX59 efficiently. Interestingly, DDX59 appears to be a doublet in western blot, and both of these two bands can be knocked down by the shRNAs as we applied. After transduction of these shRNAs through lentiviral delivery, the cells were then succumbed to multiple phenotypic analyses including cell proliferation, apoptosis, migration, and transformation assay as shown in Figures 3b–e. We found that in multiple lung cancer cell lines including H1299, Calu-1, HCC827, and SK-LU-1, deficiency of DDX59 results in significant defects in cell proliferation as shown in Figure 3b, significant reduction in anchorage-independent cell growth *in vitro* as shown in Figures 3c and d. The DDX59 knockdown efficiency for soft-agar analysis was shown in Supplementary Figure S1. These results indicated that DDX59 is essential for cancer cell proliferation and tumorigenecity *in vitro*. We also performed apoptosis assay and found that deficiency of DDX59 caused a mild increase in apoptosis as shown in Figure 3e, both of these shRNAs for DDX59 slightly increased the percentage of apoptotic cells from 7% to 13–17%.

### DDX59 promotes DNA synthesis

**Figure 4:**
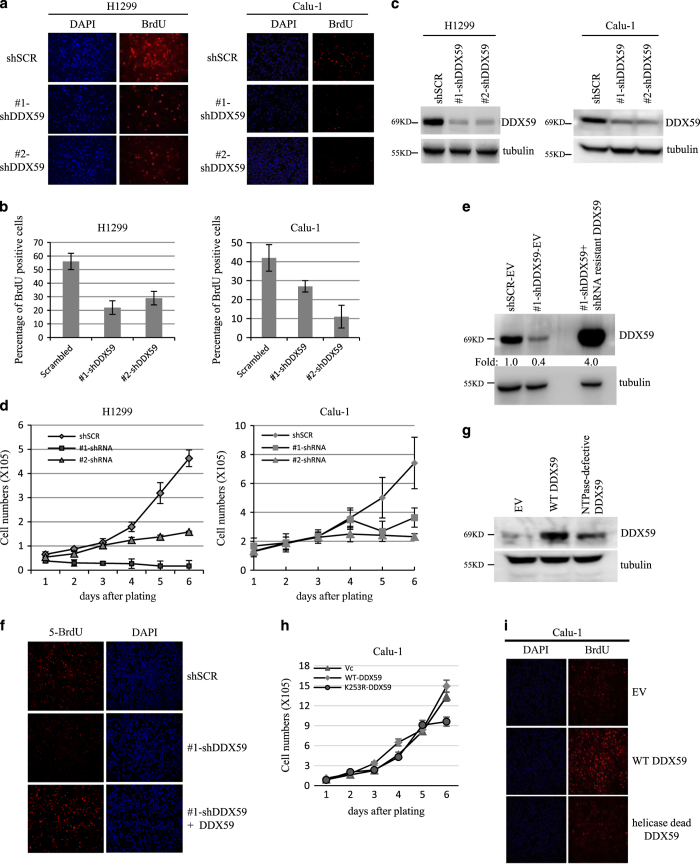
DDX59 promotes DNA synthesis and cell proliferation. (**a**) Calu-1 and H1299 cells were transduced with either shSCR or shRNA-DDX59 lentivirus. Cells were incubated with BrdU for 16 h and then stained with anti-BrdU antibody (red). Nuclei were demarked by DAPI (blue). Typical images were taken. (**b**) Quantitation of BrdU-positive cells from 50 counted nuclei in percentage. *P<0.01, *n*=3. (**c**) DDX59 proteins levels were detected by western blot analysis after lentiviral knockdown. Tubulin was used as a loading control. (**d**) The above-mentioned cells were plated onto six-well plates on the basis of equal cell numbers. Cells were then counted daily to record the proliferation curves. Bars stand for S.D. from three separate counts at one time point. (**e**) H1299 cells were first infected by lentivirus encoding either wild-type DDX59 for #1 shRNA-resistant DDX59, with empty vector for a control. Cells were then infected with lenvirus encoding #1 shDDX59, with shSCR for a control. Western blot analysis for DDX59 protein expression levels in each sample. (**f**) Above-mentioned cells were then analyzed by BrdU incorporation assay, typical images are taken. (**g**) Calu-1 cells were transduced with lentivirus encoding either wild-type DDX59 or DDX59 NTPase-defective mutant, with empty vector for a control. Western blot analysis for DDX59 protein expression levels in each sample. (**h**) Equal number of cells were then plated onto six-well plates, and counted on a daily basis for growth curve analysis. Error bars were taken from three separate counts for each sample. (**i**) Calu-1 cells were transduced with lentivirus encoding either wild-type DDX59 or DDX59 helicase-defective mutant, with empty vector for a control. Cells were then incubated with BrdU for 16 h and then stained with anti-BrdU antibody (red). Nuclei were demarked by DAPI (blue). Typical images were taken.

To investigate the mechanisms for DDX59 to promote cell proliferation, we performed BrdU incorporation assay for cells with deficient DDX59. We found that DDX59 knockdown significantly decreased DNA synthesis in both H1299 and Calu-1 cells, and influenced the growth curves for these cells as shown in Figures 4a–d. To analyze whether the reduction of BrdU incorporation could be rescued by shRNA-resistant DDX59 in cells, we first overexpressed DDX59 that was resistant to #1 shRNA-DDX59. Then, we infected the cells with shRNA to target endogenous DDX59. As shown in Figures 4e and f, the western blot data clearly show that endogenous DDX59 can be knocked down efficiently. We then analyzed the BrdU incorporation in these cells and found that when cells were infected by the shRNA-resistant DDX59, the incorporation of BrdU was rescued. These results clearly showed that #1 shRNA can knockdown DDX59 specifically in cells and DDX59 can promote DNA synthesis. The reduction in BrdU incorporation is not due to an off-target effect.

**Figure 5:**
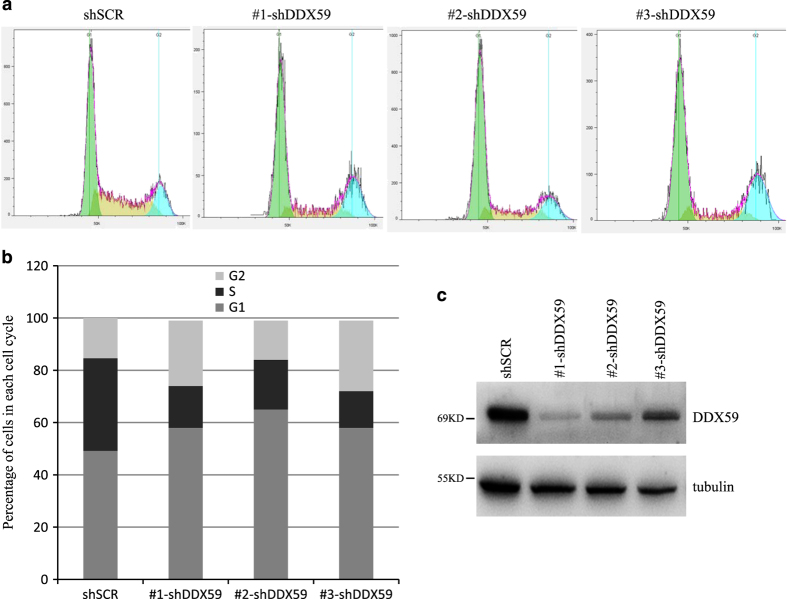
DDX59 deficiency leads to cell cycle arrest. (**a**) H1299 cells were transduced with either shSCR or shRNA-DDX59 lentivirus. Four days post infection, cells were trypsinized and fixed by 75% ethanol, stained by PI before being subjected to flow cytometry analysis. Histograms to show the cell cycle distribution after DDX59 knockdown. (**b**) Quantitation for each cell cycle phase in control and DDX59 knockdown cells. (**c**) Western blot analysis for DDX59 knockdown efficiency with tubulin as an internal control.

To verify the BrdU incorporation assay, we also analyzed DNA content by cell cycle analysis in the absence of DDX59. Cell cycle analysis allows us to evaluate whether DDX59 could promote cells entry into S phase. As shown by Figure 5, we found that in three DDX59 knockdown samples, cells demonstrated significant reduction in DNA synthesis.

We overexpressed both the wild-type and the NTPase-defective mutant of DDX59 in Calu-1 cells. We could detect an enhancement of BrdU incorporation by overexpressing wild-type DDX59 but not the mutant (Figure 4i). Likewise, we observed moderate enhancement in cell proliferation as shown in Figures 4g and h. The NTPase-defective mutant of DDX59, however, decreased the proliferation. This result implicates that the NTPase activity of DDX59 is important for DDX59 to promote DNA synthesis and cell proliferation.

### Knockdown of DDX59 reduced MCM protein levels, decreased the loading of MCM onto chromatin

**Figure 6:**
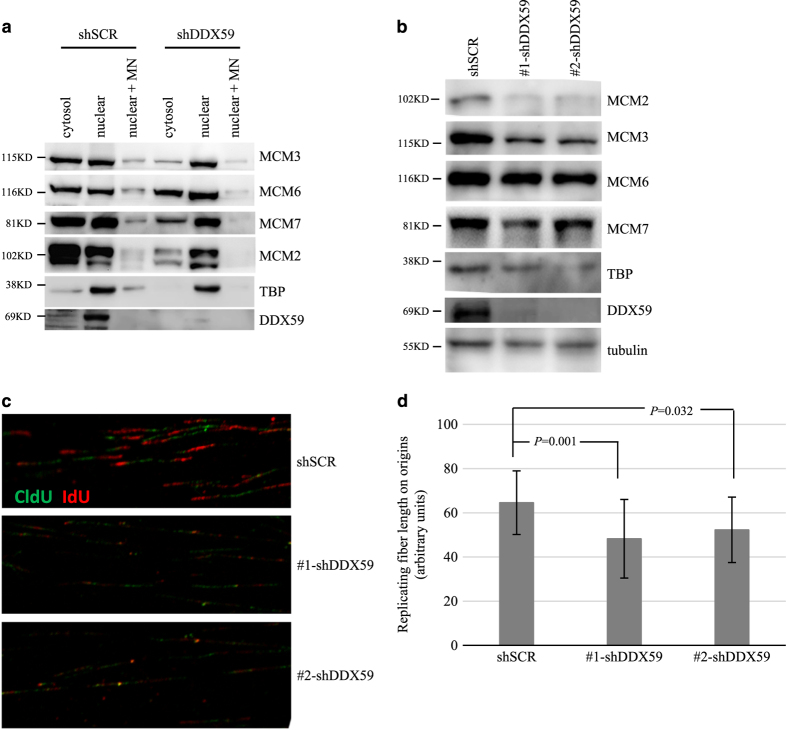
DDX59 knockdown reduces chromatin bound and total levels of MCM proteins. (**a**) H1299 cells were transduced with either shSCR or shRNA-DDX59 lentivirus. Four days post infection, cells were collected for cytosolic, nuclear soluble and chromatin-bound (by micrococcal nuclease treatment) protein fractionations. Equal proportion of each fractionation was then analyzed by western blot with the indicated antibodies. (**b**) H1299 cells were transduced with either shSCR or shRNA-DDX59 lentivirus. Four days post infection, cells were collected for total protein analysis with the indicated antibodies. (**c**) Above-mentioned cells were pulse-labeled with 50 *μ*M CldU (15 min) followed by 250 *μ*M IdU (15 min). DNA fibers were spreaded from labeled cells. Each labeled fork in the replication origins was shown as –red–green–red– segment. Typical images were shown for each sample. (**d**) The length for each replication fork was calculated from 100 tracks per condition. Data shown were the averages with S.d. bars for up to 100 tracks. *P* was calculated and shown, statistically significant difference was observed after DDX59 knockdown as compared with the shSCR control.

To analyze whether DDX59 influences the activity of MCM protein on chromatin, we first analyzed whether DDX59 would influence the binding of MCM proteins onto chromatin. We performed cell fractionation to isolate cytosolic proteins, nuclear soluble fractions as well as chromatin-binding protein fractions. As shown in Figure 6a, we found that the levels of chromatin-bound MCM proteins were decreased significantly in DDX59 knockdown cells as compared with the control cells. To study whether the reduction is due to a decrease of total MCM proteins, we performed western blot analysis as shown in Figure 6b. DDX59 protein knockdown markedly decreased the steady-state levels of MCM proteins; these include MCM2, MCM3, MCM6, and MCM7. Reduction of MCM proteins would influence the replication efficiency; to check the consequence of DDX59 deficiency on fork progression and origin activity, we performed DNA single fiber analysis. H1299 cells after DDX59 knockdown were pulsed with CldU and chased with IdU to label replicating DNA. DNA fibers were stretched and visualized after staining with antibodies. As shown in Figures 6c and d, loss of DDX59 caused marked reduction in the fork speed.

### DDX59 is required for plasmid stability, and promotes DNA replication

**Figure 7:**
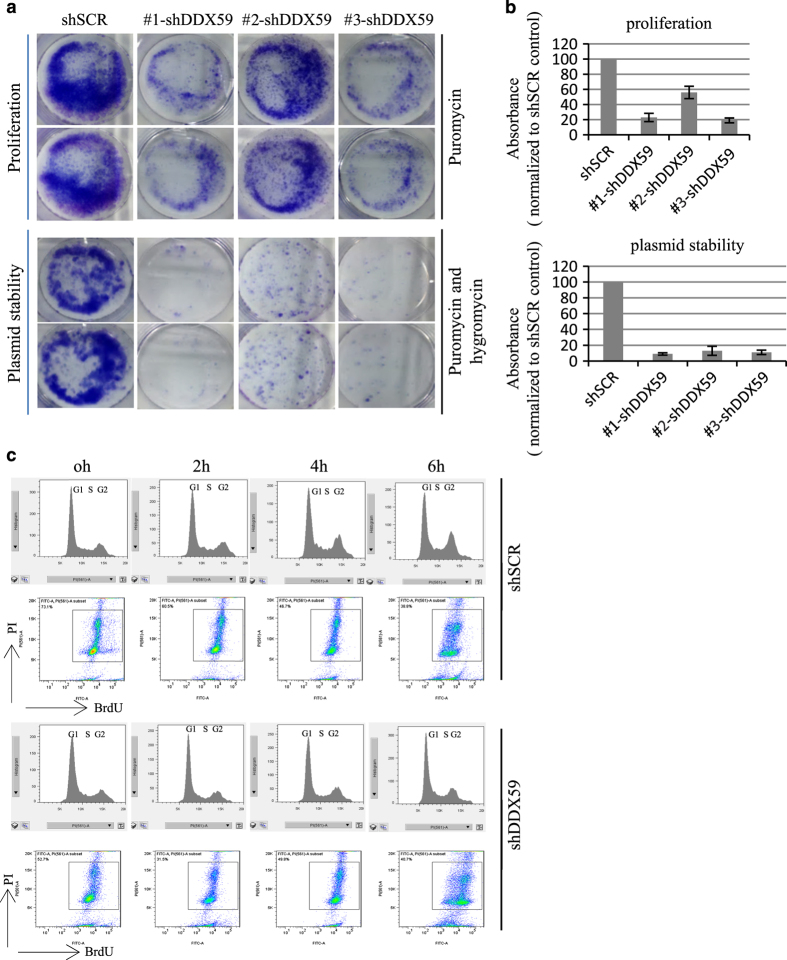
DDX59 promotes plasmid stability and cell cycle progression. (**a**) H1299 cells were transduced with lentivirus encoding either shSCR or shDDX59. Two days post infection, cells were transfected with equal amount of p220.2 plasmids, cells were then plated onto six-well dishes in equal cell numbers. For proliferation assay, cells were selected by 5 *μ*g/ml puromycin for 2 days and then changed into 2 *μ*g/ml puromycin and incubated for 1–2 weeks. For plasmid stability test, 10 times more cells were selected by 400 *μ*g/ml of hygromycin B and 5 *μ*g/ml of puromycin for 2 days, then changed into 50 *μ*g/ml of hygromycin B and 2 *μ*g/ml of puromycin and further incubated for 1–2 weeks before performing Giemsa staining. (**b**) Upper panel: quantitation of foci for the proliferation analysis samples. Bars stand for S.D. from three separate analyses. Lower panel: quantitation of foci for the plasmid stability analysis samples. Bars stand for S.D. from three separate analyses. (**c**) H1299 cells were transduced with lentivirus encoding either shSCR or shDDX59. Four days post infection, cells were pulsed with BrdU for 6 h, and then chased for 0, 2, 4, and 6 h, respectively. Cells were then analyzed by flow cytometry after incubation with anti-BrdU and PI staining. Cell cycle distribution was obtained for BrdU-positive cells only after gating.

We further analyzed the stability of an episomal plasmid in human lung cancer cells to check whether DDX59 would influence the efficiency of DNA replication. The plasmid encodes both the *EBNA1* gene and OriP that functions as a DNA replication origin.^29,30^ To be maintained in cells, OriP plasmids require both the EBNA1 protein and host cell DNA replication factors, and can link to chromosomes during mitosis and segregate to daughter cells without integration into host chromosomes. OriP plasmids replicate once-per-S phase. As this plasmid contains a hygromycin selection marker, cells can be selected by hygromycin. We transfected this plasmids into both the scrambled and DDX59 knockdown cells and then selected them in either puromycin or hygromycin/puromycin media. As shown in Figures 7a and b, we found that in DDX59 knockdown cells, few cells survived hygromycin selection, indicating that DDX59 is important in DNA replication, although the proliferation assay shows that fair amount of colonies grow on the puromycin containing media.

To further investigate how DDX59 promotes DNA replication, we first pulsed H1299 cells (either control or DDX59 knockdown cells) with BrdU for a brief time, cells were then chased in regular media for the indicated times. Cell cycle distribution was analyzed after gating BrdU-positive cells. As shown in Figure 7c, DDX59 knockdown significantly inhibits the cell cycle progression at both early G1/S and late S-to-G2 transition. The results indicate that DDX59 deficiency inhibited both the initiation and the elongation of DNA replication.

### DDX59 promotes lung cancer development in mouse xenograft

**Figure 8:**
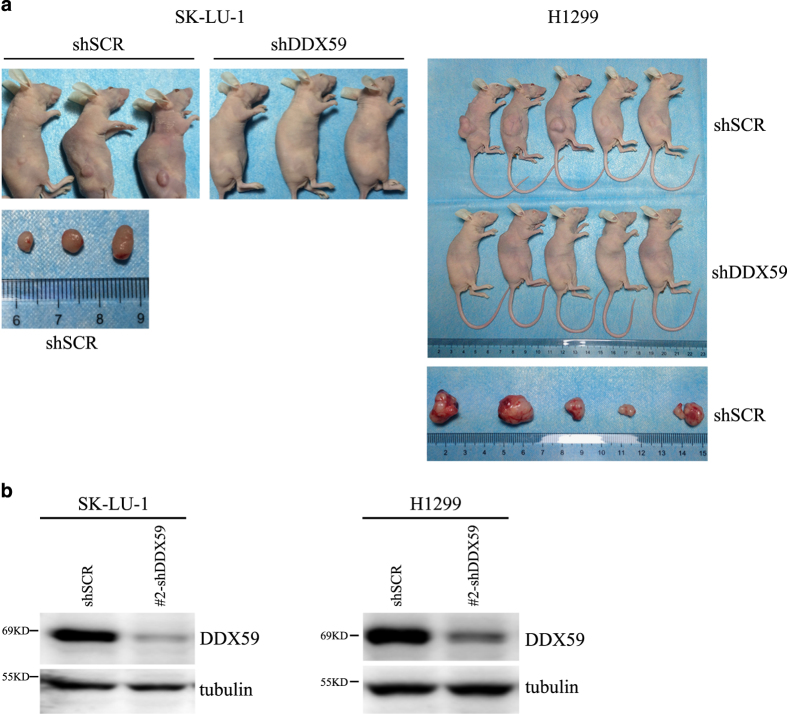
DDX59 promotes tumor growth in xenograft models. (**a**) A group of three to five NUDE mice were injected at flanks on one side with H1299 and SK-LU-1 cells, which have been transduced with either SCR or with shDDX59 (10^7^ cells per injection). Tumors were dissected after 6 weeks and images were taken before and after tumor removal. (**b**) Western blots were performed for these H1299 and SK-LU-1 cells after lentiviral infection to detect DDX59 knockdown efficiency.

To further evaluate whether DDX59 promotes lung cancer growth *in vivo*, we used the mouse xenograft models to study functions of DDX59 in tumor growth. We used lung cancer cell line for analysis. Two days after lentiviral infection of lung cancer cells, equal numbers of these cells were then injected into immunocompromised mice subcutaneously. Six weeks after injection, in the control group, which we showed the DDX59 levels were normal, cancer grew rapidly in the immunocompromised mice, whereas for the DDX59 knockdown group, no tumor could be detected as shown in Figures 8a and b. Our results clearly demonstrated that DDX59 has very important functions in promoting lung cancer development *in vitro* and *in vivo*.

## Discussion

We report that DDX59 protein is overexpressed in a significant number of human non-small cell cancers, especially in lung adenocarcinoma (up to 56%). We were able to clone a full-length DDX59 and overexpressed it in lung cancer cells. This full-length DDX59 was found to localize in the nucleus and was found to promote DNA synthesis and cell proliferation. Short hairpins targeting endogenous DDX59 resulted in decreased cell proliferation, anchorage-independent cell growth, and a marked reduction in tumor growth in xenograft models. We further found that deficiency of DDX59 caused the reduction of MCM protein levels on chromatin. These results indicated that DDX59 is playing an important role to promote DNA replication and cancer development.

From TCGA database, DDX59 appears to be highly expressed in many other human cancers. DDX59 deregulation might contribute to the development of a wide variety of cancers. It is worthwhile to further analyze the detailed molecular mechanisms for how DDX59 promotes DNA replication. In lung adenocarcinoma, EGFR and Ras mutations have been frequently detected as drivers in tumorigenesis. It would be interesting to analyze whether DDX59 can be regulated by EGFR and Ras signaling pathways. As Ras is a hard molecular drug target, analyzing the downstream signaling pathways might provide further therapeutic opportunity.

So far, this is the first report about DDX59 in human cancers and about its function in cell proliferation. The family of DEAD/DEAH box RNA helicases is a large family containing >50 multifunctional proteins involved in various steps of RNA and DNA metabolism.^6^ Many helicases perform functions that are fundamental in cell proliferation; recently, a genome-wide CRISPR gene editing found many of these RNA helicases have essential roles in maintaining hematopoietic malignant cell proliferation and survival.^14^ It is becoming apparent that many of these RNA helicase have key roles in promoting cancer cell growth and proliferation, even though the detailed underlying molecular mechanisms remain undiscovered. Our report on DDX59 function underscores the potential importance and diversity of DEAD/DEAH helicases in promoting cancer and warrants a broader evaluation of the activities of this protein family.

## Materials and Methods

### Cell culture

H1299, Calu-1, HCC827, SK-LU-1, and H23 lung cancer cell lines were maintained in RPMI-1640 medium containing 10% fetal bovine serum (FBS), 2 mM l-glutamine, and streptomycin and penicillin. BeaS2B cells were maintained in DMEM medium containing 10% FBS, and streptomycin and penicillin. WI-38 was maintained in the base medium ATCC-formulated Eagle’s minimum essential medium supplemented with FBS to a final concentration of 10%, 2 mM l-glutamine, and streptomycin and penicillin. HEK293 T cells were maintained in DMEM medium with 10% FBS and streptomycin/penicillin.

### Lentivirus production

The targeting sequences of shRNAs for human DDX59 are as follows: (5′–3′): shDDX59-1: CCCATTCAAATGCAGATGATT; shDDX59-2: GCGAGCTTTATTCGAGAGCAA; shDDX59-3: CCACAGCTTTATCGTCTGCAA; and shDDX59-4: CCTGTTATCATGCGAGCTTTA. A pLKO.1 vector encoding shRNA for a scrambled sequence was purchased from Addgene (Cambridge, MA, USA). To produce knockdown virus, 293T cells were transfected by pLKO.1 shRNA, pCMV-VSV-G, pHR Δ 8.2Δ by Lipofectamine 2000 (Life Technologies, Shanghai, China) for virus packaging. Culture supernatants were collected 24 and 48 h after transfection and then centrifuged at 2000 r.p.m. for 5 min.

### Plasmids

Full-length DDX59 cDNA (NM_001031725.4) was cloned from human primary cell cDNA library. Primers were designed to clone the open reading frame of DDX59 into pLVX-IRES-hygromycin at *Hin*dIII/*Bam*HI sites. A QuickChange site-directed mutagenesis kit (Stratagene, Shanghai, China) was used to carry out the mutation of K253 in ‘GSGKT’ motif into R. Five different shRNA sequences to target human DDX59 were designed and cloned into the pLKO.1 vector. A scrambled shRNA sequence was also cloned into pLKO.1 vector.

### Immunoblotting

Whole-cell lysates were prepared by incubation with whole-cell lysis buffer that included 0.5% NP40 and 1% SDS supplemented with HALT protease and phosphatase inhibitors (Sigma, St Louis, MO, USA). Lysates were cleared by centrifugation and protein concentration was tested by DC assay (Bio-Rad, Shanghai, China ). Lysates were boiled with SDS sample buffer, separated by SDS-PAGE, and transferred to polyvinylidene difluoride membrane (Millipore, Guangzhou, China). Membranes were blocked in 5% nonfat dry milk TBS-T (10 mmol/l Tris-HCl (pH 7.4), 150 mmol/l NaCl, 0.1% Tween 20) buffer and incubated in primary antibodies diluted in blocking buffer at 4 °C overnight. Blots were washed with TBS-T buffer and incubated with horseradish peroxidase-conjugated secondary antibodies (1 : 10 000; GE Healthcare, Shanghai, China) in blocking buffer at room temperature. Immune complexes were visualized with an enhanced chemiluminescence kit (GE Healthcare). Primary antibodies for immunodetection were sourced as follows: anti-tubulin (goat, Santa Cruz Biotechnology, Guangzhou, China), anti-DDX59 (Abcam, Guangzhou, China), anti-GAPDH (Bethyl, Beijing, China), anti-Lamin B1 (Santa Cruz Biotechnology), anti-SOD (Santa Cruz Biotechnology).

### Immunofluorescence

Cells were fixed with 10% formalin/10% methanol. Cells were then incubated with mouse anti-FLAG (Sigma) at a 1 : 1000 dilution. Goat-anti-mouse-FITC was applied to facilitate the visualization of FLAG-DDX59 protein.

### Subcellular protein fractionation

Subcellular proteins were fractionated by use of a subcellular protein fractionation kit from Pierce (Madison, WI, USA). Lung cancer cells (2×10^6^ cells) were fractionated into cytosolic and nuclear parts according to standard protocols.

### Apoptosis analysis

Apoptosis assays were performed with Vybrant apoptosis kit #2 (Molecular Probes, Shanghai, China) according to the manufacturer’s protocol.

### Cell cycle analysis

Cells were trypsinized and resuspended into phosphate-buffered saline (PBS) to generate single-cell suspension. Absolute ethanol was added dropwise into the suspension when gently vortexing to reach a final concentration of 75% of ethanol. Cells were then fixed at room temperature for half an hour or overnight at −20 °C freezer. Cells were pelleted and washed with PBS for two times and then resuspended into PI working solution (PBS containing 1% FBS, 250 *μ*g/ml of RNase A, and 30 *μ*g/ml of propidium iodide). Cells were filtered through a 35-*μ*m strainer cap (Becton Dickinson, Beijing, China) before being subjected to fluorescence-activated cell sorter analysis.

### BrdU for cell proliferation assay

Cells were previously seeded onto coverslips and at least incubated overnight after plating. Add BrdU at a final concentration of 10 *μ*M, and incubate for 18 h. Cells were then fixed at appropriate fixation buffer (2% PFA or 10% methonol/10% formalin in PBS) for 15 min at room temperature. Cells were then permeabilized with 0.3% Triton X-100 in PBS for 5 min. After washing with PBS once, cells were then treated with 1.5 N HCl at room temperature for 10 min, then blocked with 10% FBS/PBS for 1 h at room temperature. A mouse monoclonal BrdU antibody was (1 : 100) was applied onto the cells, and then incubated for 1 h at room temperature or overnight at 4 °C. Add goat-anti-mouse-Rhodamine (1 : 200) into the fixed cells and incubate for half an hour at room temperature in the dark. Wash the coverslips for five times with PBS and then mount the cells with DAPI. Cells were then ready to be visualized under fluorescence microscope.

### Focus assay

Human cancer cell lines were infected by pLKO.1 lentivirus encoding scrambled RNA or shRNA to knockdown DDX59, and cells were selected by puromycin for 2 days. Cells were then plated at a density of 10^4^ per 100-mm dish and grown for 10–20 days. Colonies were washed with cold PBS twice and fixed with 100% methanol for 10 min at room temperature. Colonies were then stained with Giemsa stain for 1 h at room temperature and washed with water before air-dried and photographed.

### Soft-agar assay

A total of 1.0×10^4^ cells were mixed in 4.0 ml 0.3% agar/DMEM/10% FBS as the top agar and plated into 60-mm plates with 4.0 ml 0.6% agar/DMEM/10% FBS as the base agar. Plates were incubated at 37 °C, checked every 3 days, and fed with 2.0 ml 0.3% agar/ DMEM/10% FBS every week. Colonies were photographed and counted 2–3 weeks later.

### Immunohistochemistry

Human tumor tissue microarrays were purchased from US Biomax (Rockville, MD, USA). This tissue microarray contains 95 cases of lung cancer. A Rabbit polyclonal anti-human DDX59 antibody (Abcam) was used at 1 : 25. Tissue slides were deparaffinized in xylene and rehydrated in a series of graded alcohols and the antigen was retrieved in Tris buffer (pH 9.0) using a steamer. The sections were then treated with 1% hydrogen peroxide in methanol for 30 min to exhaust endogenous peroxidase activity. After a 1 h pre-incubation in 10% normal FBS to prevent nonspecific staining, the samples were incubated with primary antibody at room temperature for 2 h. Standard protocol was then followed based on DAKO envision kit using polymer to amplify signals. Both immunoreactive intensity and percentage of stained cells in different areas of the same slide were analyzed according to criteria described previously.^31^ DDX59 expression was designated as 0=no staining; 1=weak; 2=moderate; and 3= strong. Additional points were scored as 1, 2, or 3 when the percentage of positive cells was <25%, 25–50%, or >50%, respectively.

### Quantitative PCR

The primers were purchased from Life Technologies. The following primers were used in quantitative PCR analysis: DDX59 (forward primer: 5′- TGTTCCCGTTGATGCTGTAG-3′; reverse primer: 5′- CTGCTCGGGACTTACTGAATG-3′); GAPDH (forward primer: 5′- TGACAACGAATTTGGCTACA-3′; reverse primer: 5′- GTGGTCCAGGGGTCTTACTC-3′). Total RNA was extracted by use of a NucleoSpin II (Clontech, Beijing, China) RNA isolation kit and was reverse-transcribed into cDNA by use of a SuperScript III first-strand synthesis kit (Invitrogen, Shanghai, China). PCRs were performed with a Step one plus thermal cycler. SYBR green mix from Bio-Rad was used for all quantitative real-time PCR analyses. Transcript quantification was calculated based on the ΔΔCT value after normalization to GAPDH values. Melt curve analysis confirmed that single products were amplified.

### DNA fiber analysis

Cells were pulse-labeled with 50 *µ*M CldU (15 min) followed by chasing with 250 *μ*M IdU (15 min). Labeled cells were collected and DNA fibers were spread in buffer containing 0.5% SDS, 200 mM Tris (pH 7.4), and 50 mM EDTA. For immunodetection of labeled tracks, fibers were first incubated with primary antibody for CldU, rat anti-BrdU (Abcam, catalog number: ab6326), and followed by goat-anti-rat-FITC secondary antibody; then with primary antibody for IdU, mouse anti-BrdU (BD Biosciences, San Diego, CA, USA, catalog number: 347580), followed by goat-anti-mouse-Rhodamine. Mouse anti-ssDNA antibody was used to assess fiber integrity. Slides were examined with a Nikon A1R Confocal Microscope (Tokyo, Japan). DNA replication efficiency was evaluated by analyzing the –red–green–red– replication origin length.

### Mouse xenograft model

NUDE female mice were purchased from Charles River Laboratories International, Inc. (Beijing, China) and received standard institutional care. They were at 5-week old at the time of surgery. For NUDE mice injection, lung cancer cells were infected as indicated. Cells were trypsinized and resuspended in PBS at a concentration of 1×10^8^ cells per ml. Five-week-old NUDE mice were injected subcutaneously with 1×10^7^ cells along their flank, with sample sizes of five mice per condition. Six weeks post infection, tumors were dissected, photographed, and weighed.
