# Supplementary material for: DDX59 promotes DNA replication in lung adenocarcinoma
Source: Cell Death Discov. 2017 Jan 9;3:16095–. doi: 10.1038/cddiscovery.2016.95 (PMC5220641; doi:10.1038/cddiscovery.2016.95)
Supplement: Supplemental Information [file cddiscovery201695-s1.doc]

Supplemental information

Table 1: Statistical analysis of DDX59 immunohistochemistry staining results for non-small cell lung cancers according to tumor subtypes and grades

| **pathology** | **DDX59 strong** | **DDX59 medium** | **DDX59 weak** | **no DDX59** |
| --- | --- | --- | --- | --- |
| normal |  |  | 3/3 (100%) |  |
| tuberculosis |  |  |  | 2/2 (100%) |
| adenocarcinoma | 17/18 (95%) | 1/18 (5%) |  |  |
| squamous cell carcinoma |  | 10/49 (20%) |  | 39/49 (80%) |
| Bronchioloalveolar carcinoma |  | 6/11 (55%) | 5/11 (45%) |  |
| adenosquamous carcinoma | 2/9 (22%) |  | 7/9 (78%) |  |
| undifferentiated carcinoma |  |  |  | 5/5 (100%) |
| small cell carcinoma |  | 3/3 (100%) |  |  |
| carcinoid |  | 1/1 (100%) |  |  |
| clear cell carcinoma |  | 1/1 (100%) |  |  |

Table 2: Matched normal, tumor adjacent normal and tumor samples from 32 different lung adenocarcinoma

| **organ** | **type** | **pathology diagnosis** | **numbers** | | **DDX59 positive samples** | **percentage of positivity** | |
| --- | --- | --- | --- | --- | --- | --- | --- |
| lung | malignant tumor | adenocarcinoma | | 32 | 18 | | 56% |
| lung | tumor adjacent | tumor adjacent tissue | | 34 | 5 | | 15% |
| lung | normal | tumor adjacent normal tissue | | 33 | 2 | | 6% |

Table 3: Statistical analysis for patient samples according to tumor stages and gender.

| **grade** | **negative** | **positive** | **percentage of positivity** |
| --- | --- | --- | --- |
| I | 8 | 4 | 33% |
| II | 3 | 6 | 66% |
| III | 3 | 8 | 73% |
| gender |  |  |  |
| F | 6 | 7 | 54% |
| M | 9 | 10 | 51% |

Figure S1


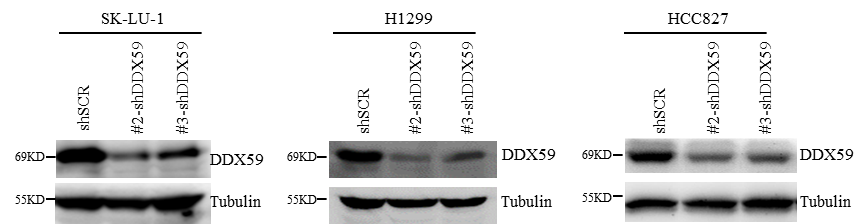


Figure S1: SK-LU-1, HCC827 and H1299 cells were transduced with either shSCR or shRNA-DDX59 lentivirus. DDX59 proteins levels were detected by western blot analysis after lentiviral knockdown. Tubulin was used as a loading control.
